# Supplementary material for: Mapping the evolution of fertility support policies in China: A content and instrumental analysis
Source: PLoS One. 2025 Oct 9;20(10):e0332137. doi: 10.1371/journal.pone.0332137 (PMC12510515; doi:10.1371/journal.pone.0332137)
Supplement: S1 Appendix — (ZIP) [file pone.0332137.s001.zip › S1 Appendix. 226 original policy documents/202-第十一届全国人民代表大会第二次会议关于2008年国民经济和社会发展计划执行情况与2009年国民经济和社会发展计划的决议(FBM-CLI-1-114470).doc]

第十一届全国人民代表大会第二次会议关于2008年国民经济和社会发展计划执行情况与2009年国民经济和社会发展计划的决议

**发布部门：** [全国人民代表大会](https://www.pkulaw.com/law/chl?Aggs.IssueDepartment=101)

**发布日期：**2009.03.13

**实施日期：**2009.03.13

**时效性：** [现行有效](https://www.pkulaw.com/law/chl?Aggs.TimelinessDic=01)

**效力级别：** [工作文件](https://www.pkulaw.com/law/chl?Aggs.EffectivenessDic=XA0105)

**法规类别：** [机关工作综合规定](https://www.pkulaw.com/law/chl?Aggs.Category=00301) [人大议事](https://www.pkulaw.com/law/chl?Aggs.Category=00106)

第十一届全国人民代表大会第二次会议关于2008年国民经济和社会发展计划执行情况与2009年国民经济和社会发展计划的决议
（2009年3月13日第十一届全国人民代表大会第二次会议通过）

　　第十一届全国人民代表大会第二次会议审查了国务院提出的《关于2008年国民经济和社会发展计划执行情况与2009年国民经济和社会发展计划草案的报告》及2009年国民经济和社会发展计划草案，同意全国人民代表大会财政经济委员会的审查结果报告。会议决定，批准《关于2008年国民经济和社会发展计划执行情况与2009年国民经济和社会发展计划草案的报告》，批准2009年国民经济和社会发展计划。

关于２００８年国民经济和社会发展计划执行情况
与２００９年国民经济和社会发展计划草案的报告
--２００９年３月５日在第十一届全国人民代表大会第二次会议上
　国家发展和改革委员会

　　各位代表：
　　受国务院委托，现将２００８年国民经济和社会发展计划执行情况与２００９年国民经济和社会发展计划草案提请十一届全国人大二次会议审议，并请全国政协各位委员提出意见。
　　一、２００８年国民经济和社会发展计划执行情况
　　过去的一年，是很不寻常、很不平凡的一年，我国经济社会发展经历了多方面难以预料、历史罕见的重大挑战和严峻考验。全国各族人民在中国共产党的领导下，深入贯彻落实科学发展观，按照十一届全国人大一次会议审议批准的国民经济和社会发展计划，克服重重困难，排除各种突发事件的干扰，全面夺取抗击南方部分地区严重低温雨雪冰冻灾害和四川汶川特大地震灾害斗争重大胜利；成功举办北京奥运会、残奥会，成功完成神舟七号载人航天飞行任务；特别是积极应对国际金融危机冲击，保持了经济平稳较快发展和社会和谐稳定。计划执行情况总体是好的。
　　（一）经济平稳较快增长
　　全年国内生产总值超过３０万亿元，增长９．０％，比世界经济平均增长率高出５个多百分点。规模以上工业增加值比上年增长１２．９％，轻、重工业分别增长１２．３％和１３．２％。全年社会消费品零售总额达到１０．８５万亿元，增长２１．６％，增速比上年加快４．８个百分点；全社会固定资产投资总额１７．２３万亿元，增长２５．５％，国内需求特别是消费需求对经济增长的拉动作用增强。
图表一、国内生产总值增长情况和国际比较（略）
经济效益继续保持增长。国家财政收入６．１３万亿元，增长１９．５％。单位国内生产总值能耗进一步降低，全年降幅为４．５９％；二氧化硫、化学需氧量排放量减少的势头得到巩固，全年分别下降５．９５％和４．４２％。万元工业增加值用水量下降７．０％。节约集约用地工作力度加大，土地利用效率继续提高。
　　（二）价格涨幅逐步回落
　　针对上半年国内食品价格上涨过快和国际市场粮食、油料价格急剧上涨对稳定物价带来的巨大压力，采取了综合性政策措施。一方面，努力保障供给。制定并组织实施扶持政策，促进粮食、生猪、油料、奶业、蛋鸡等生产发展；加强重要农副产品的紧急调运和产销衔接，缓解东北粮食、部分地区特别是灾区农产品卖难和流通不畅问题；采取财税支持、控制出口、鼓励进口、国家储备吞吐调节等综合措施，保障粮食、成品油、化肥等重要商品的市场供应和价格稳定。另一方面，加强市场监管。对粮食、食用植物油等重要商品实施临时价格干预措施。组织开展农资价格、成品油价格、医药价格和涉农收费、涉企收费、教育收费等专项检查，积极引导经营者价格自律，严厉打击囤积居奇、哄抬价格、串通涨价等违法行为。与此同时，两次提高低保补助水平，确保低收入群体的基本生活不会因物价上涨受到明显影响。进入四季度后，通货膨胀压力明显减弱，全年居民消费价格上涨５．９％；生产资料和农资价格涨幅也迅速回落，１２月份生产资料出厂价格同比下降１．９％，农资价格涨幅比最高时的７月份回落１２．４个百分点。
图表二、2008年居民消费价格指数月度变动情况（略）
（三）“三农”工作进一步加强
　　强农惠农政策力度不断加大。耕地保护工作继续加强。研究制定了进一步扶持农业和粮食生产的１０项措施，组织编制了《全国新增１０００亿斤粮食生产能力规划（２００９－２０２０年）》。在粮食收购价格、涉农补贴和主要农产品收储方面，进一步加大工作力度。三次大幅度提高粮食最低收购价，提价幅度超过２０％；中央财政用于粮食直补、农资综合补贴、良种补贴、农机具购置补贴的资金达１０３０亿元，比上年增长１倍；实施主要农产品临时收储政策，共收购稻谷１１７８万吨、玉米８５２万吨、棉花１３１万吨、油料６７万吨。
　　支农资金投入继续增加。全年中央财政用于“三农”资金达５９５５．５亿元，增长３７．９％。粮食、油料、糖料、棉花生产基地和旱作农业示范基地建设步伐加快，动植物疫病防控体系、标准化规模养殖小区、种养业良种繁育基地、农产品质量安全检验检测体系、农产品批发市场、粮油仓储及现代物流设施建设进展顺利。
图表三、中央财政用于“三农”资金的增长情况（略）
主要农副产品产量大幅增长。粮食连续５年丰收，全年总产量达到５２８５０万吨，增长５．４％，总产和单产均创历史新高。棉花产量７５０万吨，油料、糖料产量分别达到２９５０万吨和１３０００万吨，增长１４．８％和６．７％；生猪生产恢复较快，肉类总产量７２６９万吨，增长５．９％，奶业生产逐步恢复，全年牛奶产量增长３．６％；水产品产量４８９５万吨，增长３．１％。优质高产高效农业取得新进展，无公害农产品、绿色食品、有机食品和农业标准化生产加快发展，优质小麦和优质水稻所占比重分别达到６８％和７４％，比上年提高６．３个和１．９个百分点。
图表四、粮食产量及其增产情况（略）
农村生产生活条件进一步改善。病险水库除险加固、大型灌区续建配套与节水改造工程加快实施，中部大型排涝泵站更新改造规划提前完成，开工建设了一批骨干水利工程。继续加强农村水电气路建设，饮水安全工程使４８２４万农民受益，新增沼气用户５００万户，新建、改建农村公路３９．１万公里。“万村千乡”、“双百”市场工程进展顺利。
　　（四）结构调整取得重要进展
　　自主创新能力继续增强。新修订的科技进步法公布实施，国家中长期科学和技术发展规划纲要实施顺利，配套政策进一步完善。科技创新投入大幅增加，中央财政科技投入１１６３亿元，增长１６．４％。大型油气田、极大规模集成电路等１６个国家重大科技专项全面进入实施阶段，国家科技支撑、８６３、９７３等科技计划进展顺利，知识创新三期工程建设全面启动，大天区望远镜、重离子加速器等国家重大科技基础设施建成投入使用，在信息、生物和环保等领域新建了１８个国家工程中心，新认定了７６家国家级企业技术中心，支持了９０家企业技术中心创新能力建设。自主研发了百万亿次超级计算机，新支线飞机ＡＲＪ２１－７００首飞成功。
　　高技术产业和装备制造业发展加快。生物医药、卫星应用等１３个高技术产业化专项实施顺利，在信息、生物、新能源、新材料、民用航空航天等领域新认定了３７个高技术产业基地，发放了３张第三代移动通信牌照。２００８年，５４个国家高新区工业增加值增长１８．６％。振兴装备制造业的政策体系不断完善，一批重大装备和关键设备研制成功。空客Ａ３２０天津总装线正式投入运营，１０００千伏交流特高压输电试验示范线路的关键设备研制成功，兆瓦级风电设备开始批量生产。同时，继续依法淘汰落后生产能力，全年关停小火电１６６９万千瓦、小煤矿１０５４处，水泥、钢铁、电解铝等行业落后产能淘汰工作进展顺利。高耗能、高排放行业生产增幅明显放缓，钢铁、煤炭、建材等行业优势企业兼并重组步伐加快。
　　基础设施和基础产业稳定发展。新增公路通车里程近１０万公里，其中高速公路６４３３公里；新建铁路投产里程１７１９公里，增建铁路复线投产里程１９３５公里，电气化铁路投产里程１９５５公里，武汉－合肥铁路全面建成，京沪高速铁路、北京－石家庄－武汉客运专线和兰渝、贵广等西部干线及其他一批重大铁路项目开工建设；港口万吨级码头泊位新增吞吐能力３．３亿吨；港珠澳大桥前期工作取得实质性进展；新建康定、榆林、长白山等８个民用机场。福建宁德、福清与广东阳江、浙江方家山４个核电项目开工建设，两淮煤炭基地竣工验收，国家石油储备一期工程交付使用。全年原煤产量２７．９亿吨，增长４．１％，新增发电装机９０５１万千瓦。
　　服务业发展态势良好。支持服务业加快发展的政策体系不断完善，新农村综合信息服务、基础教育信息资源开发与服务、中小企业电子商务服务等信息化试点示范工程启动实施。运用服务业发展引导资金，重点支持了先进制造业服务体系、农业生产配套服务体系建设。第三产业增加值达到１２．０５万亿元，比上年增长９．５％，２００３年以来增幅首次超过第二产业。
　　区域发展协调性增强。新开工了１０项西部大开发重点工程，投资总规模达４３６１亿元，落实退耕还林后续政策，巩固退耕还林成果，实施退牧还草５２３万公顷。东北地区等老工业基地振兴步伐加快，确定了全国首批１２个资源枯竭城市，东北中小企业信用再担保机构正式成立。中部地区２６个城市比照实施振兴东北地区等老工业基地有关政策、２４３个县（市、区）比照实施西部大开发有关政策逐步落实，中部城市群发展势头强劲，“三个基地、一个枢纽”建设取得积极进展。东部地区在转变发展方式、调整经济结构、推动体制创新等方面继续走在全国前列，出台了推进长江三角洲地区改革开放和经济社会发展指导意见以及[珠江三角洲地区改革发展规划纲要](https://www.pkulaw.com/chl/13a6a2b8f9123bbebdfb.html)，经济特区、上海浦东新区、天津滨海新区开发开放继续向纵深发展。制定和实施了一批扶持老少边穷地区发展的政策性文件，区域合作和对口支援不断拓展，东部产业向中西部地区转移呈良好态势。
（五）改革开放迈出新的步伐
　　经济体制改革继续深化。农村综合改革继续深化，集体林权制度改革全面推开。公司制股份制改革和国有独资公司董事会试点稳步推进，１７家试点中央企业董事会中的外部董事都已超过半数。国有资本经营预算制度改革试点范围扩大，电信企业资产和业务重组基本完成，初步形成了新移动、新电信和新联通三足鼎立的竞争格局；航空工业体制改革取得重大进展，中航一、二集团合并重组为中国航空工业集团公司。鼓励支持和引导非公有制经济发展的配套措施不断完善。新的[企业所得税法](https://www.pkulaw.com/chl/07764981f678c159bdfb.html)得到较好落实，研究制定并发布实施了增值税转型改革方案。中国农业银行和国家开发银行改革取得重大进展，银行业的资本实力、资产质量和防风险能力有了新的提高。资源性产品价格改革稳步推进，酝酿多年的成品油价格和税费改革方案顺利实施。修改完成了深化医药卫生体制改革的意见，研究制定了２００９－２０１１年改革实施方案。综合配套改革试点工作也取得了新的进展。
　　开放型经济继续发展。对外贸易较快增长。全年进出口总额２５６１６亿美元，比上年增长１７．８％。进出口商品结构继续改善，机电产品、高新技术产品出口分别增长１７．３％和１３．１％。服务贸易快速发展。利用外资质量和水平进一步提升。完善了外商投资、借用国外贷款管理。全年吸收外商直接投资（不含银行、证券、保险领域）９２４亿美元，比上年增长２３．６％，其中，服务贸易领域实际使用外资增长２４．２％，西部和中部地区实际使用外资分别增长７９．８％和３６．４％。安排借用国外贷款２３０亿美元，比上年增长５．４％。国家外汇储备达到１．９５万亿美元。企业对外投资合作步伐加快，全年对外直接投资（非金融部分）４０６．５亿美元，比上年增长６３．６％，对外工程承包和劳务合作保持较快发展。
图表五、机电产品和高新技术产品出口比重变化情况（略）
（六）民生工作和社会事业得到加强
　　城乡居民收入继续增加、实惠增多。全年农村居民人均纯收入和城镇居民人均可支配收入分别达到４７６１元和１５７８１元，实际增长８．０％和８．４％。促进就业、以创业带动就业的政策进一步完善，城镇新增就业１１１３万人，年末城镇登记失业率４．２％。社会保障覆盖面继续扩大，全国参加城镇基本养老、城镇基本医疗、失业、工伤、生育保险人数分别新增１７５３万、９３８７万、７５５万、１６３７万和１４０６万。企业退休人员基本养老金人均每月新增１１０元，城镇居民基本医疗保险试点城市新增２２９个，新型农村合作医疗制度实现全覆盖（以县为单位）。被征地农民社会保障制度建设取得新进展。新建城镇廉租住房６３万套，通过实物配租和发放租赁补贴，又解决了１９０万户城市低收入家庭的住房困难。社区服务体系、儿童福利设施、流浪未成年人救助保护设施以及残疾人综合服务设施建设积极推进。扶贫开发力度加大、扶贫标准进一步提高，利用国家以工代赈资金建设了基本农田７．２万公顷，解决了贫困地区１９０万人、１１０万头牲畜饮水困难。安排西部地区易地扶贫搬迁人口３２万人。
图表六、城镇新增就业增加情况（略）
各项社会事业建设得到加强。城乡免费义务教育全面实施，职业教育加快发展，家庭经济困难学生资助制度逐步完善。农村义务教育办学条件进一步改善，在３５１９所农村初中学校新建６８９万平方米学生生活设施，支持７２２所中等职业学校和县级职教中心加强基础能力建设；新建改扩建１８２所特殊教育学校。启动实施高等教育“２１１工程”三期建设。高中阶段教育毛入学率比上年提高８个百分点，普通高校招生数增加４２万人，研究生增加２．７万人。基层医疗服务和计划生育设施建设加快，支持了２万余所乡镇卫生院、４３０个县医院、２０９个县中医院和３６９个县妇幼保健机构的建设；安排专门资金，支持１５９个地市级以上重点中医院、１２３１个县级和乡镇中心计划生育服务站建设，对重大传染病、地方病多发的边远地区８８００多个村卫生室给予了支持。人口自然增长率５．０８‰。广播电视村村通、乡镇综合文化站、文化和自然遗产地保护、旅游等方面的支持力度明显加大。新闻出版、文学艺术、哲学社会科学等事业繁荣发展。城乡公共体育设施建设得到加强。
（七）资源节约型和环境友好型社会建设加快
　　节能减排工作力度加大。全年累计安排４２３亿元中央财政资金，支持十大重点节能工程建设、循环经济发展、城镇污水垃圾处理设施及配套管网改造、重点流域水污染治理和节能环保能力建设等。推广节能灯６０００多万支，限产限售限用塑料购物袋取得明显成效。发布了国家重点节能技术推广目录，建筑、交通运输、公共机构节能取得新进展。实施节能减排科技专项行动，攻克了一批重点行业的重大节能减排技术，在重点领域和行业进行了大规模示范和推广应用。第二批国家循环经济示范试点顺利实施，启动了１４家汽车零部件再制造试点。经济政策的激励作用进一步强化，发布实施了节能节水环保设备所得税优惠政策，调整了不同排量乘用车的消费税税率，完善了资源综合利用税收优惠政策，出台了可再生能源电价补贴和配额交易方案。开展了省级政府２００７年节能减排目标完成情况、节能减排措施落实情况的评价考核，公布了２００７年度千家重点耗能企业节能目标责任评价考核结果，重点耗能企业能效水平对标活动进展顺利。组织开展了节能减排专项督察和环保专项执法行动。节能减排全民行动等宣传活动收到了良好社会效果。
　　生态环境保护取得新进展。塔里木河治理、黑河治理、首都水资源保护工程积极推进，石羊河流域重点治理工程全面启动；在继续推进天然林资源保护、防护林体系建设、京津风沙源治理等重点生态保护工程的同时，启动了岩溶地区石漠化综合治理。太湖水环境综合治理和松花江、淮河、辽河等重点流域水污染防治工作积极推进。三峡库区三期地质灾害防治工程顺利实施。农村环保全面启动。全国新增城市污水日处理能力１１４９万吨，城市污水处理率和城市生活垃圾无害化处理率分别达到６５％和６４％，均比上年提高２个百分点左右。燃煤电厂脱硫改造进一步推进，又有近１亿千瓦火电机组安装了脱硫设施，已占火电装机总量的６０％左右。
图表七、城市污水处理率和生活垃圾无害化处理率提高情况（略）
应对气候变化国家方案得到较好落实。发布《中国应对气候变化的政策与行动》白皮书，推动通过《应对气候变化技术开发与转让北京宣言》，清洁发展机制项目合作积极开展。
　　（八）抗击特大自然灾害取得重大胜利
　　年初，为应对南方部分地区严重低温雨雪冰冻灾害，按照中央的统一部署，各地区各部门迅速启动应急机制，全力保交通、保供电、保民生，迅速恢复了工农业生产和因灾损毁的基础设施。“５·１２”四川汶川特大地震发生后，全国上下紧急动员，人民解放军、武警部队、民兵预备役人员、公安民警和医疗卫生人员、新闻工作者、科技工作者、教育工作者、志愿者等各方面力量迅速行动，港澳台同胞及海外华人华侨慷慨解囊，国际社会积极施援，奋勇夺取抗震救灾斗争重大胜利，谱写了感天动地的英雄凯歌。从废墟中抢救出生还者８．４万人，及时收治伤病人员２９６万人次，累计救助受灾困难群众１０５８．４万人。为积极有效开展恢复重建工作，出台了[汶川地震灾后恢复重建条例](https://www.pkulaw.com/chl/0f6e2f8f567a39cdbdfb.html)，经全国人大常委会批准，设立了灾后恢复重建基金，在较短时间内编制完成灾后恢复重建总体规划和１０个专项规划，建立了“一省帮一重灾县”的对口支援机制。目前，灾后恢复重建各项规划正在抓紧实施，８４００多个重建项目已开工建设，灾区群众生产生活正逐步恢复正常。
　　在世界经济金融形势发生急剧变化、国内各种突发性困难明显增多的情况下，２００８年经济社会发展取得显著成绩，来之不易。这是党中央、国务院坚强领导、科学决策的结果，是各地区各部门密切配合、扎实工作的结果，是全国各族人民众志成城、共克时艰的结果。
　　在充分肯定成绩的同时，我们也清醒地看到，当前国际国内经济形势十分严峻复杂，保持经济平稳较快发展任务更加繁重。国际金融危机扩散蔓延，已严重冲击全球经济，波及范围之广、影响程度之深、冲击强度之大超出人们预料，主要发达经济体可能全面衰退，新兴市场经济体和发展中国家增速放缓，世界经济将会经历一个较长的低迷和调整时期，同时，全球通货紧缩压力加大，贸易保护主义抬头，不确定不稳定性因素显著增多。受国际金融危机冲击的影响，加上我国自主创新能力不强、产业结构不合理、内需外需不均衡、资源环境代价过大，以及投资消费比例关系、经济社会发展和城乡区域发展不协调等发展方式粗放的问题与体制性、结构性矛盾依然存在，经济运行困难急剧增加。特别是２００８年第四季度以来，经济增速下滑已经成为经济运行中的突出问题。一是工业生产显著放缓，１０－１２月，全国规模以上工业增加值增幅同比分别回落９．７个、１１．９个和１１．７个百分点；能源、原材料和运输需求缩减，钢材、电解铝等产品市场价格下降，产能过剩问题明显暴露。二是一些消费热点降温，全年全国商品房销售面积下降１９．７％，汽车销量增幅回落１５．１个百分点，近年来消费增长较快的其他商品需求也出现了不同程度的下降。三是对外贸易急剧下滑，去年１１、１２月份出口额分别下降２．２％和２．８％。四是部分企业经营困难，企业订单减少、销售不畅、利润缩减状况从沿海向内地、从中小企业向大企业、从外向型行业向其他行业蔓延，亏损企业增加较多。
　　与此同时，就业、“三农”、节能减排、金融等方面也存在一些突出矛盾和问题。就业再就业压力加大。全国企业用工需求明显下滑，１０－１２月城镇新增就业人数逐月下降，当月新增就业人数分别为８４万、５５万和３８万，高校毕业生、城镇就业困难人员和农民工就业形势更趋严峻。促进农业增产、农民增收的制约因素较多。农业基础设施依然薄弱，连续５年丰收后继续保持粮食生产稳定发展更加困难，农产品价格下行压力较大，在农民工返乡较多、稳定就业难度加大的情况下，保持农民收入持续增长的制约进一步突出。推进节能减排的任务更加艰巨。由于发展阶段的客观因素，节能技术服务产业发展滞后，加上目前企业经营困难加大、开工不足，导致能源利用效率和治污设施正常运营水平下降、技术改造和污染治理投入减少，“十一五”后两年节能减排的压力仍然很大。金融市场潜在风险不容忽视，资本市场信心亟待增强。部分领域市场经济秩序混乱，生产安全和食品安全重特大事故频繁发生，给人民群众生命财产造成了重大损害。针对上述问题，我们将积极采取措施，加快解决突出矛盾，努力把各种不利影响降到最低程度。
二、２００９年经济社会发展的总体要求和主要目标
　　２００９年的经济社会发展工作，要全面贯彻党的十七大和十七届三中全会精神，以邓小平理论和“三个代表”重要思想为指导，深入贯彻落实科学发展观，立足扩大内需保持经济平稳较快增长，加快发展方式转变和结构调整提高可持续发展能力，深化改革开放增强经济社会发展活力和动力，加强社会建设加快解决涉及群众利益的难点热点问题，促进经济社会又好又快发展。
　　按照上述要求，围绕保持经济平稳较快发展的首要任务，考虑速度与质量结构效益相统一、内部经济与外部经济相均衡、经济发展与社会发展相协调的需要，并与“十一五”规划《纲要》相衔接，提出的２００９年经济社会发展的主要目标是：
　　——经济保持平稳较快增长，国内生产总值增长８％左右。继续提出８％左右的经济增长目标，主要是为了防止增速下滑过多，是维护党和国家工作大局、广大人民群众切身利益的客观需要，有利于稳定社会预期、增强发展信心，有利于扩大城乡就业、增加居民收入、保持社会稳定。同时，在世界经济增长减速、外部环境复杂多变、国内矛盾明显增多的形势下，这一目标也是不低的，需要强有力的宏观政策支撑和扎实有效的工作。当然，我们所追求的是讲求质量和效益的发展，是以人为本、全面协调可持续的发展。
　　——经济结构进一步优化。自主创新力度加大，第三产业比重有所提高，产业集中度得到提升，落后产能进一步被淘汰，区域发展更趋协调，单位国内生产总值能耗、二氧化硫、化学需氧量排放量降幅进一步扩大。在国际金融危机不断蔓延、国内存在诸多矛盾和问题的大背景下，只有化挑战为机遇，变压力为动力，把保增长、扩内需、调结构有机结合起来，加快经济结构优化和企业转型升级，淘汰落后产能，整合生产要素，才能不断提高发展的质量和效益，增强产业竞争力和可持续发展能力。提出这个组合目标，就是为了引导各方面在保持经济平稳较快增长的同时，着力转变发展方式，加快调整经济结构，强化节能减排工作，努力促进企业转型升级，增强应变能力，实现又好又快发展。
　　——民生得到较好保障。城镇新增就业９００万人，实际工作中力争更多一些，城镇登记失业率不高于４．６％。农民人均纯收入和城镇居民人均可支配收入均实际增长６％。减少农村贫困人口２００万人以上。人口自然增长率控制在７‰以内。主要考虑是：越是困难的时候，越要高度关注民生，这无论是对于扩大内需、促进增长，还是增强全社会战胜困难的信心和决心，都是非常重要的。虽然当前部分企业经营困难，新增劳动力持续增加，特别是今年大学毕业生人数继续增加，就业形势非常严峻，但通过实施更加积极的就业政策，全方位增加就业岗位，加强就业服务和培训，城镇新增就业目标是可以实现的。受国内外经济环境变化的影响，农民务工难度加大，制约了现金收入的增加，生猪、棉花、大豆等主要农产品价格也存在一些不确定因素，今年城乡居民特别是农民增收形势不容乐观。但是，随着国民收入分配格局调整力度的加大，促进居民收入提高政策措施的逐步落实，农村劳动力就业渠道和农村内部增收空间的不断拓展，城乡居民收入增长的预期目标也是可以实现的。
　　——居民消费价格总水平涨幅控制在４％左右。提出这一目标，既估计了总需求增长放缓，供过于求矛盾加大，短期价格下行压力增大的因素，也考虑了稳步推进价格改革、劳动力和资源环境成本刚性上升等因素，同时也考虑了引导市场预期和稳定市场信心的需要。
　　——国际收支状况继续改善。出口保持稳定增长，进口进一步扩大，服务贸易发展加快，有效配置全球资源的能力和水平有所提高，国际竞争新优势得到加强。外贸进出口总额增长８％。利用外资的区域结构和产业结构改善，对外投资稳步增加。实现这一目标，既是保增长、保就业的现实需要，也是调结构、增效益的重要条件。尽管当前国际经济环境严峻，国际市场收缩，贸易保护主义抬头，但是，我国劳动力资源丰富、素质不断提高，技术进步加快，企业应对市场变化的能力逐步提升，在国际竞争中仍具有明显的比较优势，只要我们抓住国际分工调整的有利时机，坚持扩大对外开放，转变对外经济发展方式，走以质取胜、集约化、多元化的发展路子，就能够不断提高我国经济的国际竞争力和抗风险能力，稳定并扩大国际市场份额。
三、２００９年经济社会发展的主要任务和措施
　　今年是新中国成立６０周年，也是应对国际金融危机冲击、保持国内经济平稳较快发展和社会和谐稳定、实现“十一五”规划目标的关键一年。要着力在保增长上下功夫，把扩大内需作为保增长的根本途径，把加快发展方式转变和结构调整作为保增长的主攻方向，把深化重点领域和关键环节改革、提高对外开放水平作为保增长的强大动力，把改善民生作为保增长的出发点和落脚点，积极应对挑战，化解突出矛盾，促进经济社会又好又快发展。
　　（一）实施积极的财政政策和适度宽松的货币政策，进一步加强和改善宏观调控。把扭转经济增速下滑趋势作为宏观调控的最重要目标，进一步增强宏观调控的前瞻性、应变能力和实际效果，全力支持经济增长。
　　扩大财政支出与结构性减税相结合。（１）增加中央财政赤字和国债发行规模。２００９年拟安排中央财政赤字预算７５００亿元，比上年预算增加５７００亿元；相应增加国债发行规模。（２）减轻企业和居民税费负担。落实已出台的中小企业、房地产交易相关税收优惠和出口退税等方面政策，统一取消和停征１００项行政事业性收费。（３）优化财政支出结构。继续加大对改善民生、基础设施、灾后重建等领域的支持力度，严格控制一般性支出，压缩公务购车用车、会议经费、公务接待费、出国（境）经费等支出。严格控制党政机关楼堂馆所建设。
　　更好地发挥货币政策在促进经济增长方面的积极作用。（１）促进货币信贷供应总量合理增长。积极运用各种货币政策工具保持银行体系流动性充足。引导商业银行合理增加信贷投放，引导民间金融健康发展。（２）优化信贷结构。坚持区别对待、有保有压，加大对民生工程、“三农”、重大基础设施建设、中小企业、自主创新、技术改造和兼并重组、节能减排、区域协调发展、扩大消费、灾后恢复重建等方面的信贷支持。支持房地产开发企业合理的融资需求。（３）保持资本市场稳定健康发展。完善资本市场基础性制度，增强投资者信心。（４）加强和改进金融监管。提高金融机构风险管理水平，推进金融体系安全网建设，防范系统性金融风险。（５）保持人民币汇率基本稳定。２００９年，广义货币供应量（M2）预期增长１７％左右。
　　保持固定资产投资较快增长。（１）加大政府投资力度。从２００８年四季度到２０１０年底，中央政府拟新增投资１．１８万亿元，加上地方配套和社会投资，可形成约４万亿元的投资规模，重点用于加快保障性住房、农村民生工程、基础设施、社会事业、环境保护、结构调整和技术改造等方面建设和灾后恢复重建。２００９年，在上年末增加保障性住房、灾后恢复重建等中央政府公共投资１０４０亿元的基础上，中央政府公共投资安排９０８０亿元，比上年增加４８７５亿元。为增强地方安排配套资金和扩大政府投资的能力，国务院同意地方发行２０００亿元债券，由财政部代理发行，列入省级预算管理。（２）发挥政府投资的引导作用。拓宽民间投资渠道和领域，扩大企业债券发行规模，支持民间资本投向符合国家产业政策的领域，吸引更多的社会资金参与政府鼓励项目特别是灾后重建中的基础设施项目建设。（３）加强投资项目和资金管理。严格执行建设程序，坚决防止新一轮高耗能、高污染、低水平重复建设。进一步加强对政府投资项目的全过程监督检查，确保项目工程质量，严禁截留挪用、滞留不用和浪费建设资金，努力提高投资效益。２００９年，全社会固定资产投资总规模预期增长２０％。
　　着力扩大消费尤其是居民消费。（１）千方百计增加居民收入，提高消费能力。继续提高农资综合直补、良种补贴、农机具补贴等标准并扩大规模，提高企业退休人员基本养老金水平，提高城乡居民最低生活保障标准，提高优抚对象等人员抚恤和生活补助标准，增加居民财产性收入。（２）培育消费热点，拓展消费空间。鼓励普通商品住房消费，支持居民购买用于自住的住房，规范发展二手房市场和住房租赁市场。落实车购税减征和下乡补贴政策，稳定扩大汽车、摩托车消费。大力发展社区商业、物业、家政等服务性消费，积极开发与节假日调整相适应的旅游、文化、体育健身和网络、动漫等热点消费。加快发展电子商务。（３）完善消费政策，优化消费环境。增加消费信贷，加强城乡消费设施和服务体系建设，在全国推广“家电下乡”政策，增加热水器、计算机、空调、微波炉、电磁炉等补贴品种，加快建设“万村千乡”市场工程，推进连锁经营向农村延伸。改善城市社区便民服务设施。加强涉农、涉企、交通、教育、医药、房地产等价格收费的监督检查，维护消费者合法权益。（４）改善居民消费预期，增强消费意愿。努力消除制约消费的制度和政策障碍，适时增加对生活困难群众和家庭经济困难学生补助，减少居民扩大消费的后顾之忧，扩大即期消费。２００９年，社会消费品零售总额预期增长１４％。这一指标既考虑了近年来社会消费品零售总额持续较快增长，扩大消费政策的力度不断加大、效果逐步显现；又考虑了今年物价涨幅将比上年明显回落，以及就业压力和居民收入增长难度加大等因素对消费需求的影响。
　　搞好经济运行调节。充分发挥煤电油气运保障工作协调机制的作用。及时解决居民生活、发电和供热等重点用煤以及运输问题，做好电量安排、跨省区电能交易和优化调度，搞好成品油总量平衡和产运需衔接，确保民生、公交及公共事业等重点需求。保障灾区等重点地区和重点时段煤电油气运供应。完善应急机制和预案，增加应急物资储备，切实加强应急体系建设。
（二）巩固和发展农业农村的好形势，保障农产品有效供给、促进农民持续增收。认真落实党的十七届三中全会精神，坚持把“三农”工作作为全部工作的重中之重，把提高农民收入、夯实农业基础作为扩大内需的重要内容。（１）加大投入力度。大幅度增加国家对农村基础设施建设和社会事业发展的投入，大幅度提高政府土地出让收益、耕地占用税新增收入用于农业的比例，大幅度增加对中西部地区农村公益性建设项目的投入，新增国债也要向农村倾斜。２００９年，中央财政拟安排“三农”投入７１６１亿元，比上年增加１２０６亿元。同时，创新投入激励机制，鼓励和引导社会各方面资金投向农业农村。建成一批大中型水利骨干工程，完成一批大中型病险水库除险加固任务，加快推进大型灌区节水改造和续建配套、水源工程等重点项目建设。切实办好水、电、路、气、房五件实事，推进农村饮水安全工程建设，再解决６０００万人口的安全饮水问题，新增农村沼气用户５００万户，加快农村电网改造，积极推进农村公路改扩建工程，扩大游牧民定居工程和农村危房改造试点。建立粮食主产区利益补偿制度，加大对产粮大县一般性转移支付、财政奖励和粮食产业建设项目的扶持力度，完善粮食风险基金政策。（２）稳定发展粮食和农业生产。落实最严格的耕地保护和最严格的节约用地制度，确保粮食播种面积不少于上年。实施新增千亿斤粮食生产能力规划，加快建设国家商品粮生产基地，实施优质粮食产业工程。支持粮油高产技术大面积集成示范。强化支持粮食、油料、生猪等生产发展的各项政策，继续抓好奶业整顿和振兴工作。加强化肥价格监管。发展畜牧水产规模化标准化健康养殖，加快建设动植物疫病防控体系、农产品质量安全检验检测体系、种养业良种繁育体系、农产品批发市场、粮油仓储及现代物流设施。２００９年，力争粮食产量稳定在５亿吨水平。（３）促进农民增收。大力发展特色现代农业，发展农村二、三产业特别是农产品加工业。加快乡镇企业发展和小城镇建设，继续实施经济综合开发示范镇项目，壮大县域经济。加强农村职业教育和劳动力转移就业培训。落实国家保护农民工权益的各项政策，积极支持返乡农民工就业创业。（４）稳定农产品价格。运用提高最低收购价、储备吞吐、临时收储、跨区调运、进出口等多种调控手段，稳定粮食、食用植物油、棉花、食糖、生猪等主要农产品价格。２００９年，小麦、稻谷最低收购价平均每斤分别提高０．１１元和０．１３元。（５）深化农村综合改革。着力增强乡镇政府社会管理和公共服务职能，建立健全村级公益事业建设“一事一议”财政奖补机制。全面推进集体林权制度改革。加强土地承包经营权流转管理和服务。统筹做好国有农场体制改革和农村其他各项改革。
　　（三）加快自主创新和产业升级，进一步增强经济竞争优势。（１）组织实施重点产业调整和振兴规划。坚持标本兼治、远近结合，加强企业技术改造、推动兼并重组、淘汰落后产能、开发重大产品、创新重大技术、布局重大项目等多种措施的统筹组合，保护和发展好钢铁、汽车、船舶、石化、纺织、轻工、有色金属、装备制造、电子信息等重点产业、骨干企业、重要品牌和重要的市场份额。（２）增强自主创新能力。制定生物、软件和集成电路等产业政策。加大国有资本经营预算资金和中央政府投资力度，支持企业在生物医药、第三代移动通信、节能环保汽车等方面开展研发和推进产业化。抓紧组织实施国家重大科技专项，攻克一批具有全局性、带动性的关键共性技术。加快科技基础平台建设，推进大型天文望远镜、海洋科学考察船等重大科技基础设施建设和知识创新三期工程。继续组织建设一批工程中心、国家工程实验室和企业技术中心。大力推进企业技术改造，拟安排专项技改资金２００亿元。形成一批技术创新服务平台，加快推广应用自主创新技术和产品。加大知识产权保护力度。２００９年，研究与试验发展经费支出占国内生产总值比例力争达到１．５８％。（３）加快发展高技术产业和装备制造业。实施好信息、生物、新材料、新能源、民用航空航天等领域高技术产业化专项，集中力量推动下一代互联网、数字电视等重大高技术产业工程建设。进一步加强国家高技术产业基地和产业链建设。研究设立高技术产业创业投资引导基金。做大做强先进装备制造业。实施重大技术装备国内依托工程，落实购买和使用国产首台（套）重大技术装备的优惠政策。增强国产支线飞机自主设计与规模生产能力，推动大型核电、风电装备和高速列车等重大装备本地化。（４）促进服务业改革与发展。加强对现代服务业的支持，规范提升传统服务业。促进现代物流、信息、金融、咨询等服务业与现代制造业有机融合、互动发展。发展就业吸纳能力强和市场需求大的生活性服务业。（５）大力推进企业重组。鼓励行业龙头企业、优势企业兼并落后企业、困难企业，鼓励优势企业强强联合，鼓励关联企业、上下游企业联合重组，形成一批实力雄厚、竞争力强的大企业和企业集团。（６）采取更有力措施支持中小企业发展。落实融资担保、贴息等扶持政策，增加财政性专项资金规模，中央财政中小企业发展资金从３９亿元增加到９６亿元，健全社会化服务体系，扩大中小企业市场准入范围。（７）着力解决交通运输“卡脖子”路段，进一步优化能源结构。提高西煤东运下海和新疆煤外运能力，加强铁路客运专线、进出关通道和“三西”煤炭直达华中铁路建设。加快国家高速公路网和中西部机场建设。推进大型煤炭基地、西气东输、西电东送和城市电网改造工程建设。
（四）继续推动区域经济协调发展，缩小区域差距、优化生产力布局。（１）继续实施西部大开发战略，新开工一批重点基础设施建设项目，巩固退耕还林、退牧还草等重点生态工程建设成果。加大沿海对口支援西部力度，推动东西部共建产业园区和东部向西部产业转移。落实好支持青海等省藏区、重庆、西藏、宁夏、新疆等地区经济社会发展的政策措施。促进重点经济区域加快发展。加快提升沿边开放水平。（２）继续推动东北地区等老工业基地深化改革、扩大开放，加快传统产业技术改造和优化升级，推进粮食和农牧业生产基地建设，振兴装备制造业，发展优势特色产业，加强交通基础设施建设。完成第二批资源枯竭城市界定工作，实施资源型城市吸纳就业、资源综合利用和发展接续替代产业专项。（３）加大对中部地区优化结构和深化改革的支持力度，落实国家已经出台的政策，加快粮食、能源原材料、现代装备制造及高技术产业基地和综合交通运输枢纽体系建设。（４）推动东部地区加快经济转型、产业升级和体制创新，优先发展现代服务业、先进装备制造业、高新技术产业，注重发展海洋经济，形成新的竞争优势。（５）大力扶持革命老区、民族地区、边疆地区和贫困地区的发展，使这些地区的人民群众共享改革发展成果。（６）促进区域间生产要素合理流动和梯度转移，加快区域经济一体化进程。支持各地从实际出发，在应对外部冲击中积极发展各具特色的优势产业，培育新的经济增长点。（７）制定和实施全国主体功能区规划，明确分类调控的区域政策、各有侧重的绩效评价考核办法。
　　（五）坚定不移地深化改革扩大开放，完善有利于科学发展的体制机制。（１）稳步推进价格改革。落实好成品油价格和税费改革各项政策，完善配套措施，建立有利于科学发展的成品油价格和税收机制。继续深化电价改革，逐步完善上网电价、输配电价和销售电价形成机制。适时理顺煤电、天然气价格。推进水价改革，继续推行非居民用水超定额累进加价制度，逐步提高水利工程供非农业用水价格。（２）深化投资体制改革。健全政府投资管理体制，加快推行代建制。建立和推行投资项目后评价、重点项目公示和责任追究制度。进一步改革项目审批制度，规范核准和备案管理办法，缩减核准范围，科学界定核准权限，严格规范核准程序。（３）加大财税体制改革力度。贯彻实施新修订的增值税以及营业税、[消费税暂行条例](https://www.pkulaw.com/chl/9557bdc8c70cda8abdfb.html)，全面实施增值税转型改革。加快推进资源税费改革，统一内外资企业和个人城建税、教育费附加制度。深化预算制度改革，推进省直管县财政管理方式改革。（４）稳步推进金融体制改革。加快中国农业银行股份制改革，大力发展新型农村金融机构，继续推进政策性银行改革。发展多层次资本市场，积极稳妥推出创业板，进一步完善创业投资、股权投资和债券融资的市场制度和环境，健全市场化发行机制和发债主体自我约束机制。（５）推进医药卫生体制改革。加快建设基本医疗保障制度，建立国家基本药物制度，进一步健全基层医疗卫生服务体系，完善公共卫生机构补助政策，实施公立医院改革试点，促进基本公共卫生服务均等化。与此同时，抓紧研究制定中长期改革规划，协调推进全国和省级综合配套改革试点。继续深化国有企业、垄断行业、行政管理体制等领域的改革，支持非公有制经济适应市场变化，克服当前发展面临的困难。
　　千方百计促进外向型经济稳定发展。（１）走以质取胜、集约化、多元化的发展路子，努力保持进出口稳定增长。及时完善政策措施，切实缓解外向型企业面临的突出困难；在巩固传统市场的同时，加快开拓前景广阔的新兴市场；积极推动与有关国家和地区的自贸区建设；稳步推进加工贸易转型升级，鼓励加工贸易企业延伸产业链条和向中西部地区转移；大力发展服务贸易，抓紧落实鼓励承接国际服务外包的政策措施，积极化解国际经贸摩擦。扩大先进技术装备、关键零部件、元器件和重要能源原材料进口，增加国家战略物资和应急物资储备。（２）积极有效利用外资，提高利用外资质量。进一步改善投资环境，创新利用外资方式，引导外资投向高技术产业、先进制造业、节能环保产业和现代服务业。支持中西部地区发挥成本和资源优势承接外资转移。严格限制外商投资高耗能、高污染产业。完善国外贷款管理。２００９年，借用国外贷款安排２５８亿美元，外商直接投资预期９２４亿美元。（３）加强境外投资的规划和政策引导，积极稳妥地推进企业对外投资合作。完善与重点国家的投资合作机制，加强国际能源资源互利合作。切实防范和化解境外资产风险。综合考虑扩大境外投资的有利条件，国际金融危机对直接投资的负面影响和国内企业生产经营困难增加等因素，２００９年我国对外直接投资预期增长１３．２％。
（六）打好节能减排攻坚战，扎实推进资源节约型环境友好型社会建设。（１）加强重点工程建设。加大对十大重点节能工程、千家企业节能行动和循环经济的支持力度。强化“三河三湖”、渤海、松花江、丹江口库区及上游、三峡库区及上游、黄河中上游等重点流域、区域水污染防治，支持城镇污水处理、垃圾处理、流域综合治理等设施建设和重大环保技术示范。２００９年，城市污水处理率、城市生活垃圾无害化处理率分别达到６９％和６７％。加快北方地区供热计量改革，积极发展绿色建筑，大力推进建筑节能。加强生态建设和环境保护，稳步实施重点防护林、天然林保护、京津风沙源治理、退牧还草、岩溶地区石漠化综合治理等重点生态建设工程。（２）加快淘汰落后生产能力。严格执行产业政策，完善落后产能退出机制，建立淘汰落后产能企业名单公告制度，落实限制类和淘汰类企业实施差别电价的政策，支持中西部地区加快淘汰落后产能。２００９年，再淘汰炼铁、炼钢、造纸、电力落后生产能力１０００万吨、６００万吨、５０万吨和１５００万千瓦。（３）大力发展循环经济。完善[循环经济促进法](https://www.pkulaw.com/chl/655ad4b68f55f896bdfb.html)相关配套法规。组织编制循环经济发展规划。研究制定鼓励余热余压上网发电经济政策，研究建立循环经济发展专项资金。建立健全科学的循环经济统计制度。深化第二批国家循环经济示范试点和汽车零部件再制造试点，支持建设一批循环经济重点项目。推进灾区建筑废弃物等资源化。加强资源综合利用，全面推行清洁生产。万元工业增加值用水量预期下降５．６％，工业固体废物综合利用率达到６５．９％。（４）完善法规政策。出台《固定资产投资项目节能评估和审查条例》，加快制定或修订高耗能产品能耗限额强制性国家标准和主要用能产品强制性能效标准。研究提出支持高效节能空调、节能电机、节能环保型汽车推广和促进节能服务产业发展的经济政策。扩大能效标识范围和节能节水产品政府采购范围。落实企业购买节能节水环保设备所得税和节能环保小排量汽车消费税优惠政策。组织开展节能管理师试点。继续加强脱硫电价监管，完善需求侧电价管理制度。继续开展排污权有偿使用和交易试点。（５）严格考核监管。进一步完善节能减排目标责任考核办法。组织开展省级政府和千家企业２００８年节能目标完成和措施落实情况考核。加强节能监察能力建设。强化环境保护与管理。以保障群众饮水安全为重点，依法查处各类环境违法行为。促进燃煤电厂脱硫和城市污水处理厂等治污设施正常运营。继续开展节能减排全民行动。（６）全面实施应对气候变化国家方案。积极推进中央和省级应对气候变化能力建设，建立健全各级管理机构。编制与实施省级应对气候变化方案，组织开展低碳经济试点，推动清洁发展机制项目的国际合作，加强减缓和适应气候变化技术研发。
　　（七）加大改善民生力度，着力解决涉及群众利益的热点难点问题。（１）实施更加积极的就业政策。充分发挥服务业、劳动密集型产业、中小企业、非公有制经济吸纳就业的重要作用。把促进发展、扩大内需与千方百计增加就业结合起来，在安排投资和确定重大建设项目时，注重发挥对就业的带动作用；大力支持自主创业、自谋职业，促进以创业带动就业；通过灵活的就业机制缓解高校毕业生就业压力，鼓励高等院校运用科研专项吸收毕业生参与研究，鼓励高校毕业生到城乡基层社会管理、公共服务岗位就业和参军入伍，到民营企业和中小企业就业；进一步开发公益性就业岗位，重点做好就业困难群众、零就业家庭和受灾地区劳动力就业援助工作；切实处理好节能减排、淘汰落后生产能力中涉及的职工安置问题。加强人力资源服务体系、职业培训体系和创业服务体系建设，继续做好农村劳动力转移就业服务基础设施建设试点工作；积极扶持农民工返乡创业；关注宏观经济变化对就业的影响，做好失业动态重点监测工作。（２）加快完善城乡社会保障体系。扩大城镇基本养老保险、基本医疗保险以及失业、工伤和生育保险覆盖面，推进基本养老保险省级统筹，加快扩大做实个人账户试点，制定全国统一的养老保险关系转续办法和农民工参加基本养老保险办法。巩固和完善新型农村合作医疗制度。加强社保公共服务设施建设。提高城市和农村低保补助水平，调整优抚对象等人员的抚恤和生活补助标准，推动建立城乡居民最低生活保障等与物价变动相适应的动态调整机制。２００９年，城镇参加基本养老保险人数预期增长４．２％。（３）加快实施城乡保障性安居工程。大规模开展廉租住房建设，在配套资金、土地供应等方面予以保证，中央政府今年拟安排财政资金４３０亿元，用于补助低保住房困难家庭的实物廉租住房建设。积极发展公共租赁住房。加快城市棚户区和国有林区、垦区、煤矿棚户区改造工程，完成搬迁维修采煤沉陷区民房任务。在中西部地区扩大农村危房改造试点，加快游牧民定居工程实施进度，改善农村人居环境。（４）加强收入分配调节。建立健全企业职工工资正常增长机制和支付保障机制，推进国有企业收入分配制度改革，强化垄断行业工资分配的调控，加快收入分配监测体系建设。（５）整顿和规范市场经济秩序。抓好与民生密切相关的各类价格和收费监管，严厉打击各种违法违规生产经营行为；深入开展食品安全专项整治，健全并严格执行产品质量安全标准；建立权责利相对应的法律追究惩治体系。（６）全力维护社会稳定。加强社会管理和安全生产工作。着力抓好城乡一体化进程中的社会管理；健全针对巨灾和突发公共事件的应急预案和政策措施；强化流动人口服务和管理工作。落实生产安全责任制，坚决遏制重特大安全事故发生。加强社会治安综合治理。（７）继续做好扶贫开发工作，扩大易地扶贫搬迁、以工代赈规模。实行新的扶贫标准，对农村低收入人口全面实施扶贫政策。
（八）加强社会事业建设，努力增强经济与社会发展的协调性。（１）优先发展教育。实施全国中小学校舍安全工程，推进农村中小学标准化建设。继续实施中西部农村初中校舍改造工程，加强中西部地区中小学教师培训，完善进城务工人员子女教育的相关政策措施，推进义务教育均衡发展。加快发展中等职业教育，特别要重点支持农村中等职业教育，２００９年起对中等职业学校农村家庭经济困难学生和涉农专业学生实行免费。继续推进县级职教中心、中等职业学校基础能力建设和中西部地区特殊教育学校建设。实施高等教育“２１１工程”三期和“９８５工程”。２００９年，高中阶段教育毛入学率预期达到７７％，普通高校计划招收本专科生６２９万人，研究生４７．５万人。（２）着力加强公共医疗卫生。进一步完善公共卫生服务体系，增强重大疾病防治能力以及突发公共卫生事件预测预警和处置能力。健全城乡基层医疗卫生服务体系，继续支持农村三级卫生服务网络和基层计划生育服务体系建设，推进城市社区卫生服务机构建设。加强重点中医院和中医临床研究基地建设。将农村部分计划生育家庭奖励扶助标准由人均６００元提高到７２０元，全面推进“少生快富”试点工作，在全国农村实施孕产妇住院分娩补助政策和出生缺陷早期干预措施，对农村妇女开展妇科疾病定期检查。（３）大力发展文化、广播影视、新闻出版、体育等事业和旅游业。加强公共文化服务体系特别是基层公共文化设施建设。推进乡镇综合文化站建设，继续实施广播电视村村通、“西新工程”和农村电影放映等工程。繁荣发展哲学社会科学。加强文化和自然遗产地保护，做好公共博物馆、纪念馆免费开放工作并完善相关机制。积极发展新兴文化产业。继续实施农民体育健身工程，促进基层体育事业健康发展。加强旅游基础设施建设，完成红色旅游重点景区建设任务。
　　（九）扎实推进灾后恢复重建工作，为灾区群众安居乐业打好基础。（１）全面推进民生工程恢复重建。按照民生优先、统筹安排、保证重点的原则，加快城乡住房和教育、卫生等民生工程的恢复重建。优先保障灾区群众的住房建设，到年底基本完成农村居民住房重建任务，全面启动城镇住房重建工作。安排好学校、医院等公共服务设施的恢复重建，确保今年底９５％以上的学生都能在永久性校舍中学习。严格执行强制性标准规范，既确保建成安全、牢固、群众放心的建筑，又避免超标准铺张浪费。（２）加快交通、通信、能源、水利、流通等基础设施恢复重建。全力做好灾区公路的保通工作，优先安排干线公路和通县公路的恢复重建，抓好乡村公路建设。加快电网和供电设施恢复重建。加强地质灾害防治和生态修复，搞好受损堤防、水库的除险加固和堰塞湖治理。（３）结合群众就业推进产业重建。把安排群众就业与恢复重建结合起来，尽可能多地吸纳灾区劳动力参与到恢复重建工作中。重点支持就业容量大的企业恢复生产和重新建设。（４）加强组织协调，推动灾后恢复重建规划的顺利实施，进一步做好对口支援工作，优化对口支援方案，及时研究解决恢复重建中的重大问题。
　　继续加强内地与香港、澳门两个特别行政区在经贸、科技、教育、文化、卫生、体育等领域的交流合作，加快推进与港澳地区货物贸易的人民币结算试点，采取有力措施支持香港、澳门抵御国际金融危机冲击、促进经济发展。抓住两岸关系改善的历史机遇，建立具有两岸特色的经济合作机制，开展经济合作，扩大两岸直接“三通”，支持海峡西岸和其他台商投资相对集中地区的经济社会发展。
　　各位代表，做好２００９年经济社会发展各项工作，任务艰巨，意义重大。我们将更加紧密地团结在以胡锦涛同志为总书记的党中央周围，高举中国特色社会主义伟大旗帜，坚持以邓小平理论和“三个代表”重要思想为指导，深入贯彻落实科学发展观，认真接受全国人大的指导和监督，虚心听取全国政协的意见和建议，开拓进取，扎实工作，为实现经济社会发展目标，促进又好又快发展而努力奋斗！

© 北大法宝：（[www.pkulaw.com](https://www.pkulaw.com/)) 专业提供法律信息、法学知识和法律软件领域各类解决方案。北大法宝为您提供丰富的参考资料，正式引用法规条文时请与标准文本核对。
欢迎查看所有[产品和服务](https://www.pkulaw.com/helps/2.html)。法宝快讯：[如何快速找到您需要的检索结果？](https://www.pkulaw.com/helps/70.html)[法宝](https://www.pkulaw.com/helps/69.html)V6有何新特色？

扫描二维码阅读原文

**原文链接：**<https://www.pkulaw.com/chl/5281abf8e9c2bd93bdfb.html>
